# End Results in Gall Bladder Surgery

**Published:** 1913-09

**Authors:** E. L. Griffin

**Affiliations:** Atlanta, Ga.


### Journal-Record of Medicine

Successor to Atlanta Medical and Surgical Journal, Established 1855
and Southern Medical Record, Established 1870

**OWNED BY THE ATLANTA MEDICAL JOURNAL COMPANY**

PUBLISHED MONTHLY

*Official Organ Fulton County Medical Society, State Examining Board, Presbyterian Hospital, Atlanta, Birmingham and Atlantic Railroad Surgeons' Association, Chattahoochee Valley Medical and Surgical Association, Etc.*

EDGAR BALLENGER, M. D., Editor
BERNARD WOLFF, M. D., Supervising Editor
A. W. STIRLING, M. D., C. M., D. P. H.; J. S. HURT, B. Ph., M.D.
GEO. M. NILES, M. D., W. J. LOVE, M. D., (Ala.); Associate Editors
E. W. ALLEN, Business Manager

**COLLABORATORS**

DR. W. F. WESTMORLAND, General Surgery
F. W. McRAE, M. D., Abdominal Surgery
H. F. HARRIS, M. D., Pathology and Bacteriology
E. B. BLOCK, M. D., Diseases of the Nervous System
MICHAEL HOKE, M. D., Orthopedic Surgery
CYRUS W. STRICKLER, M. D., Legal Medicine and Medical Legislation
E. C. DAVIS, A. B., M. D., Obstetrics
E. G. JONES, A. B., M. D., Gynecology
R. T. DORSEY, Jr., B. S., M. D., Medicine
L. M. GAINES, A. B., M. D., Internal Medicine
GEO. C. MIZELL, M. D., Diseases of the Stomach and Intestines
L. B. CLARKE, M. D., Pediatrics
EDGAR PAULIN, M. D., Opsonic Medicine
THEODORE TOEPEL, M. D., Mechano Therapy
R. R. DALY, M. D., Medical Society
A. W. STIRLING, M. D., Etc., Diseases of the Eye, Ear, Nose and Throat
BERNARD WOLFF, M. D., Diseases of the Skin
E. G. BALLENGER, M. D., Diseases of the Genito-Urinary Organs

VOL. LX. ATLANTA, GA., SEPTEMBER, 1913. NO. 6

### END RESULTS IN GALL BLADDER SURGERY.

BY DR. E. L. GRIFFIN, ATLATNA, GA.

The end results of gall stone surgery is not different from that of other organs of the abdominal cavity, namely, that a given surgeon will in this class of work achieve both gratifying and disappointing results, this being due to the widely varying pathological states in which the viscus and its ducts are found.

Gall bladder work differs from that of the appendix in that it is rare indeed for a person to consent to a surgical operation at the first attack of gall stone colic or cholecystitis.

In simple inflammation of the gall bladder where the walls are only moderately thickened and the contents of the organ only partially disorganized, there is no operation in surgery in which more pleasing results are obtained, and this statement will hold good even when the bladder has been long diseased and having one or many stones as inhabitants of it. We often find that after many years of morbidity due to gall stones the walls themselves are fairly healthy and, by simply cleaning out the bladder and instituting efficient drainage we effect a cure and the patient returns to normal.

On the other hand, in any elderly person, where there has been a gradual distention of the bladder with steadily increasing jaundice in which no pain has been manifested, the outlook for a complete recovery following any kind of an operation is meagre indeed. Between these two extremes there is a vast pathological field which will put to the utmost test the judgment and skill of the surgeon.

In analyzing the latter division, the following are some of the conditions to be found together with the results to be expected to ensue upon surgical intervention:

In the first place, in old or even middle-aged people who have been the victims of long standing gall bladder disease and its attending ills, such as gastric, intestinal and hepatic disturbances, the cellular metabolism seems to be impaired in such a way that after a complete and thorough operation they sometimes fail to return to the standard of health to be desired. They tire easily and complain of their old friend, biliousness and indigestion, but at the same time are free of their former gall bladder pains, etc.

Another condition found occasionally is the strawberry gall bladder and in this we have a serious state of affairs to combat. When the operator finds the lining membrane of the bladder exactly simulating a nearly full-ripe strawberry, the bladder should be completely removed, as this type will almost surely continue to ooze blood and come to a fatal termination; thus while it is thought that the removal of the gall bladder as a spectacular display has about run its course, its excision under these conditions is fully justified.

Occasionally an operator will find that the fundus of the organ is either gangrenous or so nearly so that it is thought wise to remove it. If such a condition is found half of it may be cut away and the remainder invaginated around the drainage tube and without attaching it to the abdominal wall get a satisfactory result. Personally it is thought unnecessary to attach the fundus to the belly wall for the reason that it is likely to become anchored there to the discomfort and danger of the individual.

When stones are lodged in the ducts their removal is accomplished with difficulty oftentimes and on account of this and other factors one or more may be left behind and it is an almost forlorn hope to expect them to work out. Neither can the operator side-step the point under discussion by saying that the stones have reformed; for it is thought that no individual can ever grow two sets of gall stones, and, therefore, it behooves the surgeon to get all the stones while in the belly or else the operation will be a perfunctory one and a discredit to surgery. With a complete clearing of the ducts of stones and where no great organic change has occurred in the ducts, such as stricture, perforations into the head of the pancreas, etc., a patient possessing ordinary stamina and habits may reasonably expect ordinary good health and digestion as his pay for the hazard of the operating table.

A new element in gall stone work has developed within the past few years in relation to gall bladder disease and pellagra. It has been my melancholy fate to operate upon two pellagrins, and in one it was impossible to demonstrate that the individual had a gall-bladder though it was searched for by myself and another surgeon for more than one hour. The area where it should have been was filled with free bile and bile could be seen welling up through the mass of adhesions, but no bladder to be found. The patient made good recovery from the operation but died with full symptoms of pellagra six months later. The second case was similar but the bladder could be easily demonstrated though it was greatly shriveled. The suggestion is made without enthusiasm that the poison of pellagra in some way dries up the viscus.

This paper is read without text book reference and is based on personal observation. The writer having had no experience with gall stone illeus or of stones lodging in the papilla, that phase of the subject will not be referred to here.

827 Austell Building.

